# Archives of Ophthalmology and Otology

**Published:** 1870-09

**Authors:** 


### Archives of Ophthalmology and Otology.

We have received the No. 2 of the Archives of Ophthalmolgy and Otology. This number, as the first, contains many valuable original contributions to Ophthalmic and Aural surgery. Many of them are also most beautifnlly illustraed with chromo-lithographic plates and wood cut illustrations. The following is the contents of the second number :—

Purulent Otitis Media, caused by the Nasal Douche, and accompanied by Double Hearing.—By H. Knapp, of New York. The Influence of Spectacles on the Optical Constants and Visual Acuteness of the Eye.—By H. Knapp. Large Cyst of the Iris, cured by operation.—By H. Knapp. A Case of Extirpation of a Cancroid Growth of the Inner Canthus and Eyelid.—Blepharoplasty by Sliding Flaps.—By H. Knapp. On the Measurement of the Prominence of the Eye.—By P. Keyser of Philadelphia. A New Form of Wire Snare for the Removal of Aural Polypi, modified from that of Wilde.—By C. J. Blake, of Boston. Report of a Case of Detachment of the Choroid from the Sclerotic, after an Operation for Cataract, with Partial Loss of Vitreous Body. —By George Reuling, of Baltimore. The use of Acetic Acid in Affections of the Conjunctiva and Cornea.—By B. A. Pope, of New Orleans. Anæsthesia of the Cornea, and Concurrent Diminution of the Action of Atropia on the Iris.—By J. S. Hildreth, of Chicago. Contributions to Physiological Optics.—By B. A. Pope of New Orleans. Recovery of Complete Nervous Deafness. —By S. Moos, of Heidelberg. Melanotic Sarcoma of the Ciliary Body and adjoining Choroid.—By H. Knapp. On the Pathology of the Vitreous.—By H. Pagenstecher, of Wiesbaden. Injury of Left Eye; Sympathetic Ophthalmia of the Right; Loss of Vision in the Eye secondarily affected! Vision retained in the Injured Eye.—By Thomas R. Pooley, of New York. Serous Accumulations in the Tympanum.—By S. Moos. On the Mechanism of the Ossicles of the Ear.—By Albert H. Buck of New York. Historical and Critical Remark concerning the Deafness following Meningitis Cerebro-Spinalis —By S. Moos. Sudden Hemorrhage into the Right Tympanum, accompanying Angina Diphtheritica. Protracted Recovery.—By S. Moos. A Case of Idiopathic Diphtheria of the External Meatus.—By S. Moos. Cysticercus Intra-Ocularis.—By J. Hirschberg, of Berlin. Granulation Tumors of the Iris —By J. Hirschberg, of Berlin, and Dr. Steinheim, of Bielefeld. Do the eyes perform any Rotation on th e Optic Axes in Lateral Inclinations of the Head. —By Joseph Aub, of Cincinnati. The Mechanism of the Organ of Hearing.—By H. Kaiser, of Dieburg. The Diagnosis of Intra-Ocular Sarcomata.—By O. Becker, of Heidelberg. A Preliminary Notice on the Anatomy and Physiology of the Eustachian Tube.—By S. Moos. Explanation of Plates. General Alphabetical Index of Volume I.

